# Discriminating cognitive status in Parkinson’s disease through functional connectomics and machine learning

**DOI:** 10.1038/srep45347

**Published:** 2017-03-28

**Authors:** Alexandra Abós, Hugo C. Baggio, Bàrbara Segura, Anna I. García-Díaz, Yaroslau Compta, Maria José Martí, Francesc Valldeoriola, Carme Junqué

**Affiliations:** 1 Medical Psychology Unit, Department of Medicine, Institute of Neuroscience, University of Barcelona, Barcelona, Catalonia, Spain; 2 Centro de Investigación Biomédica en Red sobre Enfermedades Neurodegenerativas (CIBERNED), Hospital Clínic de Barcelona, Barcelona, Catalonia, Spain; 3 Movement Disorders Unit, Neurology Service, Hospital Clínic de Barcelona, Institute of Neuroscience, University of Barcelona, Barcelona, Catalonia, Spain; 4 Institute of Biomedical Research August Pi i Sunyer (IDIBAPS), Barcelona, Catalonia, Spain

## Abstract

There is growing interest in the potential of neuroimaging to help develop non-invasive biomarkers in neurodegenerative diseases. In this study, connection-wise patterns of functional connectivity were used to distinguish Parkinson’s disease patients according to cognitive status using machine learning. Two independent subject samples were assessed with resting-state fMRI. The first (training) sample comprised 38 healthy controls and 70 Parkinson’s disease patients (27 with mild cognitive impairment). The second (validation) sample included 25 patients (8 with mild cognitive impairment). The Brainnetome atlas was used to reconstruct the functional connectomes. Using a support vector machine trained on features selected through randomized logistic regression with leave-one-out cross-validation, a mean accuracy of 82.6% (p < 0.002) was achieved in separating patients with mild cognitive impairment from those without it in the training sample. The model trained on the whole training sample achieved an accuracy of 80.0% when used to classify the validation sample (p = 0.006). Correlation analyses showed that the connectivity level in the edges most consistently selected as features was associated with memory and executive function performance in the patient group. Our results demonstrate that connection-wise patterns of functional connectivity may be useful for discriminating Parkinson’s disease patients according to the presence of cognitive deficits.

Cognitive impairment is a frequent non-motor manifestation of Parkinson’s disease (PD), and can already be detected in 19 to 24% of newly-diagnosed patients^1^,^2^. In the long term, up to 80% of patients develop dementia^3^,^4^, but the time from disease onset to dementia is highly variable^3^,^5^. Among different negative prognostic factors, the presence of cognitive impairment that does not significantly interfere with daily life activities, that is, mild cognitive impairment (MCI), portends a higher risk of conversion to dementia^3^,^6^,^7^,^8^.

Different neuroimaging techniques have the ability to characterize the pathological substrates of neurodegenerative diseases such as PD^9^. One such technique, resting-state functional magnetic resonance imaging (fMRI), has emerged as a promising approach to describe the functional architecture of the brain^10^. Resting-state fMRI allows mapping the synchronized activity (functional connectivity) between regions spanning the entire brain, describing the macroscopic *functional connectome*^11^.

As MRI techniques quickly evolve, there is growing interest in their potential to define biomarkers for risk, diagnosis, prognosis, and treatment response in several pathological processes. In order to reach this end, neuroimaging studies should focus on multivariate models with predictive power at the single-subject level rather than on describing differences between patient and control groups using univariate approaches^12^. Supervised machine-learning algorithms, which classify unknown data sets through models built from a set of labelled training data, are suitable candidates for this task^12^,^13^.

Previous resting-state fMRI studies have demonstrated that cognitive impairment in PD is associated with altered functional connectivity^14^,^15^,^16^,^17^. It is unclear, however, whether patterns of resting-state connectivity allow distinguishing individual PD patients based on the presence of cognitive deficits, and whether these connectivity patterns can be used as biomarkers for worse cognitive prognosis in PD.

In the present study, we use resting-state fMRI data to classify a group of PD patients according to the presence or absence of mild cognitive impairment (MCI). We test the hypothesis that multivariate edge-wise patterns of resting-state functional connectivity allow classifying PD patients according to cognitive status, using resting-state fMRI data combined with a detailed neuropsychological assessment. Specifically, our goal in the current study was to assess whether functional connectivity abnormalities could reliably distinguish patients with MCI from patients without it. To this end, we employed a supervised machine-learning algorithm combined with functional connectomics data, cross-validated through a leave-one-out procedure, and validated using an independent sample.

## Results

### Subjects

Table 1 shows the sociodemographic, clinical, and head motion characteristics of the training sample. In this sample, 27 PD patients (38.6%) were classified as having MCI (PD-MCI) and 43 (61.4%) as not having MCI (PD-nonMCI). By definition, PD-MCI patients showed significantly worse cognitive performance than PD-nonMCI, and also had longer disease duration and higher dopaminergic medication dosage. No significant cognitive differences were found between healthy controls (HC) and PD-nonMCI. No significant intergroup differences were observed for age, years of education, sex, or hand dominance. A significant group effect was observed for mean interframe head rotation; post-hoc testing showed that both PD subgroups had higher head rotation than controls, with no significant differences between them.

Supplementary Table 2 shows demographic, clinical, and head motion characteristics for the validation sample. In this sample, 8 patients (32.0%) were classified as PD-MCI and 17 (68.0%) as PD-nonMCI. No significant age (p = 0.944) or education (p = 0.884) differences were observed between PD patients in the training and validation samples. Likewise, no significant differences between the two samples were observed in MMSE (p = 0.884), mean attention/working memory (p = 0.804), executive function (p = 0.463), visuospatial/visuoperceptual (p = 0.545), or memory (p = 0.893) scores. The proportion of male to female patients was higher in the validation sample (χ^2^ = 3.681, p = 0.055).

### Classification – leave-one-out cross-validation

The cross-validated classification procedure correctly predicted overall subjects’ groups with a mean accuracy of 82.6% (3.9% standard deviation (SD)). The mean AUC was 0.88 (0.01 SD). Mean classification accuracies in both PD-nonMCI and PD-MCI groups were also 82.6% (with respective SD of 3.5% and 4.3%).

**Figure 1:**
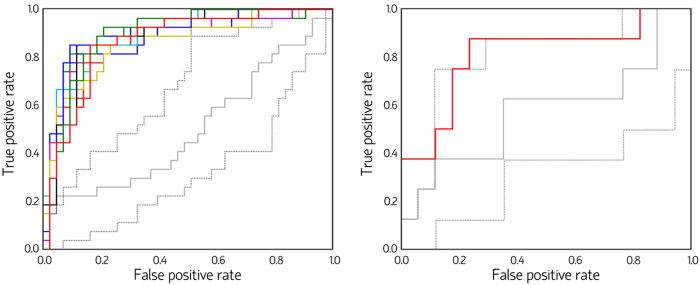
Classification receiver operating characteristic curves (ROC). Color curves indicate actual-model ROC (Left panel: ROC corresponding to the 10 actual-model feature selection repeats; Right panel: ROC corresponding to the classification of the validation sample). Example curves obtained from the null-model randomization procedures are also shown (left: reshuffling of group membership; right: reshuffling of the edges used as features). The central solid gray line depicts an ROC with an area under the curve close to the median of all permutations, whereas the lower and upper dotted gray lines represent ROC with areas under the curve close to percentiles 2.5 and 97.5, respectively, of the random distributions.

Statistical significance of the classification accuracy in the training sample was established through two null distributions (similar to ref. 18). In the first null model, group membership was randomly reshuffled between PD-nonMCI and PD-MCI, and the SVM procedure was repeated, in a total of 1,000 iterations. Each subject *y* was classified using the features identified in ≥8 of the 10 cross-validation repeats in which *y* was the test sample (*i.e.*, feature selection was not repeated). The null model had a mean accuracy of 50.0% (9.5% SD), and was outperformed by the actual model in all permutations (p < 0.001) (see Fig. 1). In the second model, group membership was also randomly reshuffled 1,000 times. Feature selection through RLR, followed by SVM classification, was repeated at each reshuffle. This null model showed a mean accuracy of 49.6% (1.34% SD) (p = 0.002), with mean AUC of 0.50 (0.15 SD).

The results above indicate that the set of edges identified through RLR allow classifying patients according to group membership. However, they cannot rule out that the classification performance was influenced not only by the different cognitive profiles between PD-MCI and PD-nonMCI. PD-MCI patients had significantly higher LEDD and HY scores than PD-nonMCI subjects. Although not significant at the α-threshold specified, the higher age in PD-MCI compared with PD-nonMCI subjects might also have biased the performance of the classifier. We therefore repeated the classification procedure using a subsample of PD-nonMCI (n = 26) matched for age, education, disease duration, sex distribution, head motion parameters, LEDD, and UPDRS and HY scores with the PD-MCI subjects (Supplementary Table 1). The classification procedure using L2 regularization correctly predicted group membership with an accuracy of 81.5% (4.8% SD; p < 0.001), with a mean AUC of 0.90 (0.02 SD). An average of 91.9% (2.8% SD) of PD-nonMCI and 71.1% of PD-MCI (6.7% SD) subjects in this matched sample were correctly classified.

### Classification – independent validation sample

In this step, a SVM model was trained using the whole training sample and the 21 edges most consistently chosen as features in the randomized logistic regression (RLR) + leave-one-out cross-validation (LOOCV) procedure. This model was then used to classify the 25 subjects in the validation sample. The classifier achieved an AUC of 0.81, with 80% of subjects (n = 20) correctly classified, including 7 patients in the PD-MCI group (87.5%) and 13 PD-nonMCI patients (76.5%).

In order to test whether the classification accuracy could be ascribed to the specific features used to train the SVM model, we generated a null distribution by randomly reshuffling the location of the 21 feature edges in the brain network and repeating the SVM procedure 10,000 times. The null model showed a mean accuracy of 55.0% (10.9% SD) and mean AUC of 0.57 (0.13 SD), and was significantly less accurate than the actual model (p = 0.006).

### Functional connectivity analysis

#### Edges used as features for classification

**Figure 2:**
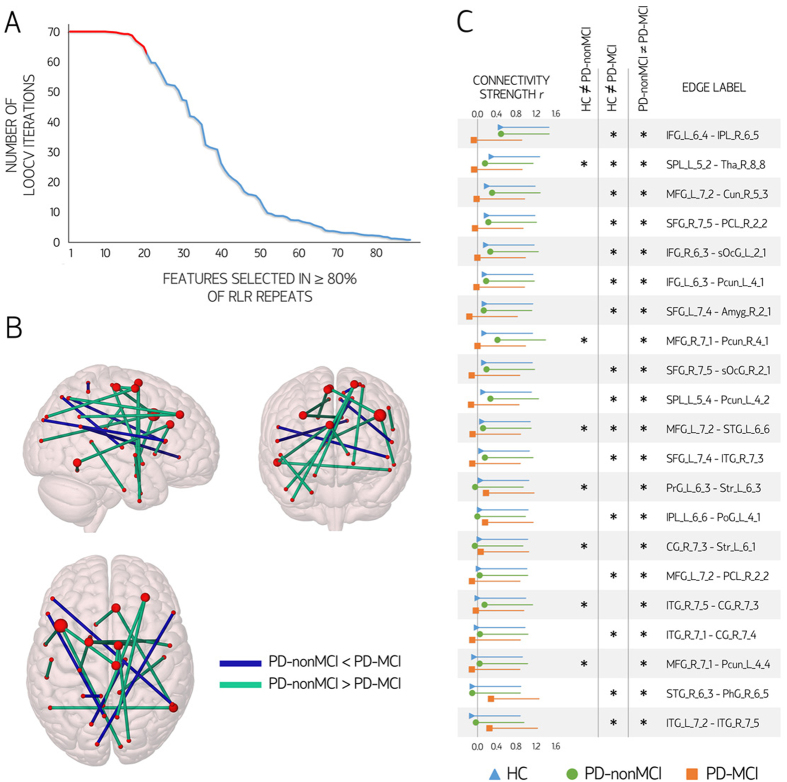
Edges used as features in the classification procedure. Panel (A) shows the distribution of the 89 edges selected as relevant features in all randomized logistic regression (RLR) feature selection repeats. Twenty-one edges were selected in ≥80% of the leave-one-out cross-validation (LOOCV) iterations (red segment). Panel (B) depicts the location of the 21 edges most consistently selected as relevant features to discriminate Parkinson’s disease patients without mild cognitive impairment (PD-nonMCI) from patients with mild cognitive impairment (PD-MCI). Brain nodes are scaled according to the number of edges connected to them. Panel (C) shows the mean strength values and standard deviation of the 21 most consistent features according to group. Significant pairwise intergroup differences in edge strength (increases or decreases; p < 0.05 with false-discovery rate control) are marked with an asterisk. Labels of pairs of nodes connected by each edge are shown (see Supplementary Table 3). *r:* connectivity strength (Pearson’s correlation coefficient). *HC:* healthy controls. *Amyg:* Amygdala; *BG:* basal ganglia; *CG:* Cingulate gyrus; *IFG:* Inferior frontal gyrus; *IPL:* Inferior parietal lobule; *ITG:* Inferior temporal gyrus; *LOcC:* lateral occipital cortex; *MFG:* Middle frontal gyrus; MVOcC: medioventral occipital cortex; *PCL:* Paracentral lobule; *Pcun:* precuneus; *PhG:* Parahippocampal gyrus; *PoG:* Postcentral gyrus; *PrG:* Precentral gyrus; *SFG:* Superior frontal gyrus; *SPL:* Superior parietal lobule; *STG:* Superior temporal gyrus; *Tha:* Thalamus. Brain plots were created with Surf Ice (https://www.nitrc.org/projects/surfice/).

Figure 2 shows the 21 edges, connected to 34 nodes, most frequently selected as features across RLR and LOOCV iterations. In all 21, mean values were significantly different between PD-nonMCI and PD-MCI (all p < 0.001, surviving FDR correction). To test whether these edges displayed a predominant intra/inter-regional distribution or whether they were homogeneously distributed across the brain, we labeled them according to the regions connected (frontal, temporal, medial temporal, insula, cingulate, parietal or occipital; see Supplementary Table 3). We then compared the observed edge distribution to a null model consisting of randomly reshuffling the 21 edges across the brain network in 10,000 iterations. No topographic pattern was significantly overrepresented.

Seven nodes were connected to more than one of the 21 stable feature edges. Five such nodes were located in the frontal lobes, namely, in the right middle frontal gyrus (inferior frontal junction), left middle frontal gyrus (dorsal Brodmann area 9/46), left superior frontal gyrus (dorsolateral Brodmann area 6), right superior frontal gyrus (medial Brodmann area 6), and right paracentral lobule (Brodmann area 4, lower limb). The remaining two nodes connected to more than one edge used as a feature were the right inferior temporal gyrus (Brodmann area 37, ventrolateral), and the right pregenual cingulate cortex (Brodmann area 32).

#### Correlates of the edges used as features for classification

In the set of 21 edges most consistently selected as features, connectivity strength was significantly reduced in the PD-MCI group when compared with PD-nonMCI in 16 edges; in 13 of these, connectivity strength was also significantly reduced when compared with HC (p < 0.05, FDR-corrected). In the five remaining edges, connectivity was significantly stronger in PD-MCI than in PD-nonMCI; in three cases, strength was significantly higher in PD-MCI than in HC (p < 0.05, FDR-corrected) (see Fig. 2). To assess the clinical and cognitive correlates of the 21 most-consistent edges, we created two variables: the first by averaging the connectivity strength in the 16 edges with lower mean values in PD-MCI than in HC/PD-nonMCI; and the second by averaging strength in the 5 edges with higher values in PD-MCI than in the other groups.

Initially, we correlated these mean values with cognitive scores, age, education, LEDD, disease duration, and UPDRS and HY scores in the whole PD group. As the values in these edges differed significantly between PD-nonMCI and PD-MCI, their mean values were also expected to correlate with the clinical and cognitive variables that were different between the two patient groups (*i.e.*, cognitive scores, LEDD, disease duration). Indeed, mean values in the 16 weakened edges correlated significantly with executive function (r = 0.53, p < 0.001), memory (r = 0.49, p < 0.001), and visuospatial/visuoperceptual scores (r = 0.27, p = 0.022), as well as with LEDD (r = −0.29, p = 0.016), disease duration (r = −0.42, p < 0.001), and HY scores (r = −0.30, p = 0.011), all surviving FDR control. In order to evaluate whether the strength in these connections was significantly associated with these clinical and cognitive variables while taking the others into account, we entered cognitive (attention/working memory, executive, memory, visuospatial/visuoperceptual) and clinical measures (HY scores, LEDD, disease duration) as independent variables in a multiple linear regression model. Mean connectivity strength in the 16 weakened edges, defined as the average correlation coefficient across these connections, was entered as the dependent variable. This analysis showed significant effects for executive function (standardized β = 0.40, p < 0.001) and memory scores (standardized β = 0.29, p = 0.005), as well as for disease duration (standardized β = −0.35, p = 0.008), all surviving FDR correction.

No correlations between the mean values in the 5 edges with increased values in the PD-MCI group survived FDR correction. No significant correlations were observed between mean connectivity values and cognitive/sociodemographic variables in the HC group. Also, no significant correlations were observed between mean connectivity values and head motion parameters, either in the HC or the PD patient group.

#### Characterization of whole-connectome intergroup connectivity differences

Comparison of mean whole-connectome strength (average of correlation coefficients across all network edges) showed a significant group effect (F = 5.390, p = 0.006). Post-hoc testing showed that both PD-nonMCI and PD-MCI had significantly reduced connectivity compared with HC (respectively, p = 0.024 and p < 0.001, surviving FDR control). No significant differences were observed between PD subgroups (p = 0.068) (see Supplementary Figure 2).

**Figure 3:**
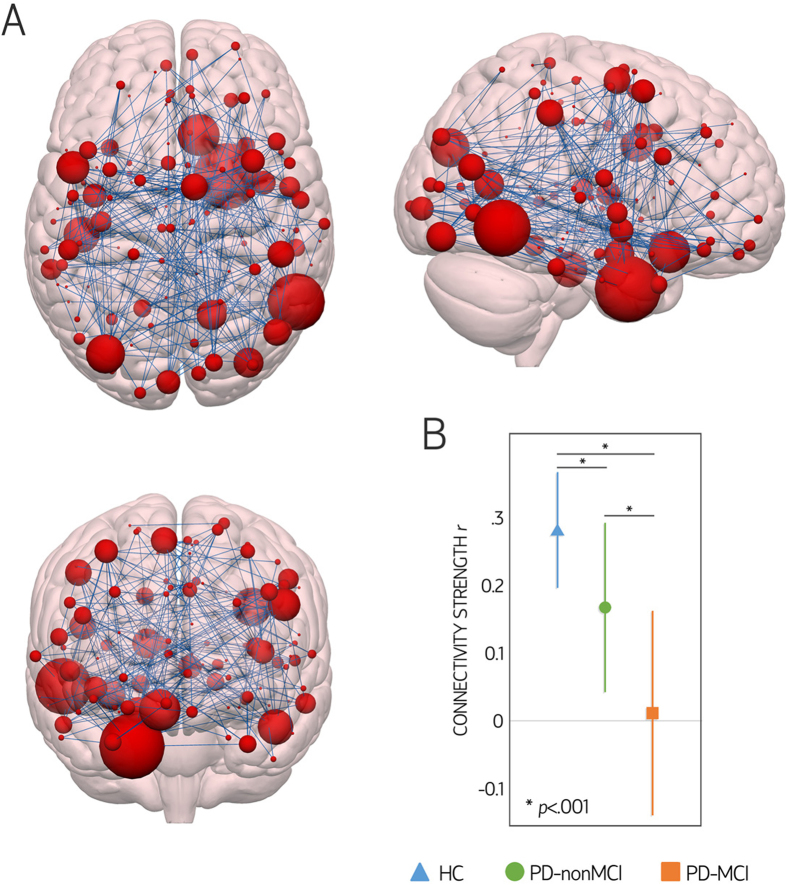
Comparison between healthy controls (HC) and Parkinson’s disease patients with mild cognitive impairment (PD-MCI) using network-based statistics (NBS). (**A**) Schematic representation of the component consisting of 235 edges considered significantly different between groups (p < 0.05, family-wise error corrected). Brain nodes are scaled according to the number of edges in the significant component to which they are connected. Panel B shows the means and standard deviations of the connectivity strength of the 235 significant connections according to group. No significant differences were found between HC and Parkinson’s disease patients without mild cognitive impairment (PD-nonMCI) patients, or between PD-nonMCI and PD-MCI patients using NBS. *r:* connectivity strength (Pearson’s correlation coefficient). Brain plots were created with Surf Ice (https://www.nitrc.org/projects/surfice/).

Intergroup comparisons using *network-based statistics* (NBS) revealed significant connectivity reductions in PD-MCI compared with HC. Specifically, a component comprising 235 edges connected to 120 nodes displayed reduced connectivity strength between these groups (p < 0.017) (see Fig. 3, and Supplementary Figures 3 and 4). Analysis of regional distribution of these 235 edges showed that occipital-temporal (n = 22) and occipital-frontal (n = 24) edges were overrepresented in the component of reduced connectivity in PD-MCI compared with HC (both p < 0.001, surviving FDR control).

No significant differences were observed between HC and PD-nonMCI or between PD-nonMCI and PD-MCI with NBS, either at the predetermined component-defining threshold of F = 11, or at the lower exploratory thresholds. Mean values across the 235 edges in the component of significant connectivity reductions in PD-MCI compared with HC, on the other hand, were significantly lower in PD-nonMCI compared with HC, and in PD-MCI compared with PD-nonMCI (F = 40.173, p < 0.001; all post-hoc p < 0.001, surviving FDR control; see Fig. 3).

Correlation analyses between cognitive/clinical/demographic variables and mean strength values across the 235 edges comprised in the component of significant connectivity reductions in PD-MCI compared with HC showed significant correlations with executive function (r = 0.36, p = 0.002), memory (r = 0.32, p = 0.006), and visuospatial/visuoperceptual scores (r = 0.27, p = 0.023); HY scores (r = −0.24, p = 0.044); LEDD (r = −0.32, p = 0.007); and disease duration (r = −0.34, p = 0.004). No significant correlations were observed between mean strength values across these 235 edges and cognitive variables in controls.

## Discussion

Using resting-state functional connectomes and supervised machine-learning, we have achieved a high accuracy in classifying PD patients with and without MCI. Classification was performed using a linear SVM trained on features selected through a stability selection method, cross-validated using LOOCV, and validated in an independent sample. These results suggest that multivariate connection-wise patterns of functional connectivity disruption may be useful for discriminating PD patients according to cognitive status. Our findings also corroborate previous descriptions of altered functional connectivity in PD associated with cognitive impairment.

We have observed significant connectivity alterations in PD patients, especially in the PD-MCI group, in agreement with previous findings^14^,^17^,^19^. In contrast with these previous studies, which focused on the description of group differences, we assessed whether resting-state functional connectivity could discriminate PD patients at the individual level. Classification performance was high and significantly better than predicted by chance. In the training sample, a balanced mean accuracy of 82.6% in both PD-nonMCI and PD-MCI groups was achieved. In the held-out validation sample, 76.5% of PD-nonMCI and 87.5% of MCI subjects were correctly classified.

A relationship between cognitive and motor deficits, previously described in PD^20^,^21^, was not observed in our patient group. Nonetheless, PD-MCI patients in our training sample had longer disease duration and higher equivalent dopaminergic medication daily intake than PD-nonMCI subjects. The classification results described above could therefore have been influenced by different aspects of the disease, beyond cognitive decline, that can be associated with altered resting-state functional connectivity in PD^17^,^22^,^23^,^24^. We therefore performed an additional cross-validated classification procedure including a PD-nonMCI subsample matched with the PD-MCI group for disease severity and dopaminergic medication intake, and with more similar age distributions. This procedure also yielded a high accuracy in classifying PD-MCI and PD-nonMCI, giving further support to our hypothesis that connectivity patterns allow discriminating PD patients with cognitive impairment from those without it. To our knowledge, this is the first study to demonstrate this.

In previous studies addressing PD through machine-learning approaches, structural MRI parameters have been used to discriminate PD from essential tremor^25^, or from progressive supranuclear palsy^26^,^27^,^28^. In the context of Alzheimer’s disease (AD), a few recent studies used resting-state functional connectivity measures to discriminate patients with AD from MCI patients and from controls. The accuracy in discriminating AD patients from healthy subjects using functional connectivity measures is often reported to be higher than 90%^29^,^30^. Distinguishing cognitively unimpaired controls from MCI subjects, on the other hand, appears to be more difficult, and reported discrimination performances are usually low^31^. Using network edges as features, Challis *et al*. (2015) achieved an accuracy of 75% in discriminating HC from MCI patients, and of 97% in discriminating amnestic MCI from AD patients, suggesting that the discrimination between HC and MCI is harder than the distinction between MCI and dementia^29^. Schouten *et al*. (2016), using network edges defined through full correlations as features, discriminated mild-to-moderate AD patients from HC with an accuracy of 76.7%. Other authors used graph-theoretical parameters as input to the classification procedure. Dyrba *et al*. (2015) achieved an accuracy of 74% in separating AD patients from HC^32^. Khazaee *et al*. (2016) separated HC from both MCI and AD patients with an accuracy of 87%; of 98% when separating AD patients from HC and MCI subjects; and of 72% when discriminating MCI from HC and AD subjects^30^.

While the functional connectivity reductions observed in PD-MCI subjects compared with HC occurred between all major brain regions, regional distribution analysis showed that they disproportionately involved occipital-temporal and occipital-frontal connections. Alterations in occipital and posterior temporal areas have been described in PD-associated cognitive impairment using different neuroimaging modalities. Abe *et al*. (2003) described that occipital hypoperfusion correlated with visual cognitive deficits using N-isopropyl-p-[^123^I]iodoamphetamine single-photon emission computed tomography^33^. More recently, Garcia-Garcia *et al*. (2012) found occipital and inferior parietal hypometabolism in association with more severe cognitive impairment and dementia using fluorodeoxyglucose positron emission tomography^34^. Finally, in structural MRI studies, PD-MCI patients have been shown to have occipital as well as posterior parietal and temporal cortical thinning^35^,^36^. Our findings add to this putative relationship between posterior cortical alterations and cognitive impairment in PD. Nonetheless, the features that best discriminated PD-nonMCI from PD-MCI, according to the feature extraction approach used, showed no clear regional predilection. The nodes connected to more than one of the 21 edges most consistently used as features, on the other hand, were mostly located in frontal or anterior cingulate regions. Regression analyses showed that connectivity strength in these edges was associated with performance in memory and executive functions, but not with attention/working memory or visuospatial/visuoperceptual performance, when taking cognitive and non-cognitive clinical variables into account.

Cognitive deficits in PD are heterogeneous and involve different neuropsychological functions^37^,^38^. It has been hypothesized that cognitive abnormalities in PD can be broadly categorized into two types: *frontostriatal deficits*, associated with dopaminergic imbalances; and *posterior cortical deficits*, which include visuospatial/visuoperceptual impairments^39^,^40^. Frontostriatal deficits mainly include attention and executive impairments^41^. These cognitive functions, in turn, are thought to rely on frontostriatal circuits as well as on the interaction of large-scale cortical networks that mainly engage frontal, insular and parietal regions such as the dorsal attention network and the frontoparietal networks^42^,^43^,^44^. According to some authors, certain aspects of the memory deficits shown by PD patients are also attributable to executive dysfunction^45^. Although the exact etiologies of posterior cortical deficits are not clear, they may be related to cortical α-synucleinopathy, cholinergic deficits, and cortical Alzheimer’s-type pathology^40^,^46^,^47^,^48^. Posterior cortical deficits are especially relevant since they appear to increase the risk of future dementia, more so than frontostriatal impairments^39^,^40^,^47^. Other studies, however, have related the presence of frontal atrophy and hypometabolism to the occurrence of MCI and dementia in Parkinson’s disease^49^.

The main interest in the development of neuroimaging biomarkers sensitive to cognitive impairments in neurodegenerative diseases lies in their potential ability to predict cognitive decline, so as to identify at-risk patients who could benefit from early, possibly disease-modifying treatments^50^. Considering the above, it is noteworthy that the edges that most reliably discriminated PD-nonMCI from PD-MCI patients most frequently connected to frontal nodes. Future longitudinal studies are necessary to clarify whether the connections that discriminated PD-nonMCI from PD-MCI in the present study are able to reliably predict cognitive decline over time, or whether they mainly reflect the cognitive differences between these groups that do not herald worse cognitive outcomes. Identifying the connectivity substrates of different types of cognitive deficit in PD, and how these substrates predict future cognitive deterioration, can also help determining whether the current approach for defining MCI in PD is appropriate, or whether it could benefit from giving differential weights to posterior cortical and frontostriatal deficits (see also ref. 39).

Neuroimaging techniques such as fMRI have been traditionally used to study the pathophysiology of brain disorders by comparing patient groups with healthy cohorts^51^,^52^. More recently, a growing number of studies have attempted to develop prognostic/diagnostic tools through neuroimaging^12^,^51^. In order to become clinically useful, the findings of such studies need to demonstrate reproducibility and generalizability^53^,^54^. As is the case with other scientific fields, however, many fMRI studies have failed to replicate^53^,^55^. The use of different acquisition sequences^56^, image preprocessing steps^53^,^57^ as well as flexibility in data collection, analysis, and reporting^55^ are some potential culprits to this “replication crisis”. In our study, we assessed the generalizability of our findings through the use of an independent validation sample. The validation data set, however, was obtained using identical fMRI acquisition parameters (except for the acquisition length), image preprocessing pipeline, and analytical methods. Also, despite recent efforts to standardize the cognitive assessment in PD patients^58^, neuropsychological tests currently used are still very different among research groups, with potentially different sensitivity to types of cognitive impairment. As such, we cannot assume that the set of discriminating network connections identified in our study would generalize to data sets obtained using other neuropsychological batteries or analytical tools.

## Limitations

Some limitations to the present study should be pointed out. While care was taken in the feature selection and cross-validation procedures to increase the generalizability of the classifier, a larger sample would have allowed the use of cross-validation procedures with smaller variance than LOOCV. Another common caveat in resting-state fMRI studies in PD are the effects of head motion on connectivity estimates. Despite the rigorous exclusion criteria applied, PD patients in the training sample still showed higher motion than HC. No significant differences, however, were observed between PD-MCI and PD-nonMCI; also, connectivity values in the edges used as features did not correlate with head motion parameters. Additionally, quality control measures indicate that image preprocessing successfully reduced the dependency of signal change on head motion. These observations suggest that motion is unlikely to have had a significant impact on the classification procedure. Finally, it is known that the parcellation scheme used can have a large impact on the characteristics of the reconstructed functional connectome^59^. Although optimal approaches have not been determined, parcellations based on connectivity profiles, such as the one used in the present study, appear to be useful for determining brain areas with similar connectivity profiles and, therefore, with likely similar function^60^.

## Conclusions

In this study, we demonstrate for the first time that multivariate patterns of resting-state functional connectivity alteration can be used to distinguish non-demented PD patients according to cognitive status through a machine-learning approach. As such, data extracted from functional connectomes have the potential to serve as biomarkers for severity of cognitive impairment in PD.

## Methods

### Participants

Two subject samples were included in this study. The first, used as a training sample, was initially comprised by 91 non-demented PD patients and 41 HC. The second sample, used only as a validation set, initially included 30 non-demented PD patients from a different cohort. All patients were recruited from the Parkinson’s Disease and Movement Disorders Unit, Hospital Clínic de Barcelona. HC were recruited from individuals who volunteered to participate in scientific studies at the Institut de l’Envelliment, Universitat Autònoma de Barcelona. The inclusion criterion for patients was the fulfillment of the UK PD Society Brain Bank diagnostic criteria for PD. Exclusion criteria were: Mini-Mental State Examination scores < 25 or dementia according to Movement Disorder Society criteria; Hoehn and Yahr (HY) score > III; significant neurological, systemic or psychiatric comorbidity; pathological MRI findings other than mild white matter (WM) hyperintensities; mean interframe head motion ≥ 0.3 mm translation or 0.3° rotation; and maximum interframe head motion ≥ 1 mm translation or 1° rotation.

One control, nine patients from the training sample, and five patients from the validation sample were excluded due to excessive head motion. Two controls were excluded due to microvascular WM changes. From the training sample, one patient was excluded due to ventricular dilatation that prevented adequate normalization to Montreal Neurological Institute (MNI) standard space, one due to a small cerebral infarction, and 10 due to psychiatric comorbidities (mainly severe depressive symptoms). The final training sample consisted of 70 PD patients and 38 HC matched for age, sex, and years of education. Fifty-seven patients and 35 HC overlapped with the cohorts analyzed in refs 14 and 61. The final validation sample included 25 PD patients, matched to the training sample for age and years of education.

All patients except one were taking antiparkinsonian drugs, consisting of different combinations of levodopa, cathecol-O-methyl transferase inhibitors, monoamine oxidase inhibitors, dopamine agonists, and amantadine. All assessments were done while patients were under the effect of their usual medication (*on* state). Levodopa equivalent daily dose (LEDD) was calculated as suggested by Tomlinson *et al*.^62^ Motor disease progression and severity were evaluated using HY and Unified Parkinson’s Disease Rating Scale motor section (UPDRS) scores. The study was approved by the institutional ethics committee (Clinical Research Ethics Committee of the Hospital Clínic de Barcelona), all subjects provided written informed consent to participate (IRB00003099), and all methods were performed in accordance with the relevant guidelines and regulations.

### Neuropsychological assessment

All subjects underwent a thorough neuropsychological assessment addressing cognitive functions frequently impaired in PD. The same battery was administered to all subjects in both training validation samples. Neuropsychological tests were grouped by cognitive function following a scheme similar to that used previously^14^,^61^.

*Attention/working memory:* backward minus forward digit spans, Stroop Color-Word Test interference scores. *Executive function*: Trail-Making Test part A minus part B scores, and phonemic fluency scores (words beginning with “P” produced in 60 seconds). *Visuospatial/visuoperceptual functions*: Benton’s Visual Form Discrimination and Judgment of Line Orientation tests. *Memory*: Rey’s Auditory Verbal Learning Test total learning and 20-minute free recall scores.

Z-scores for each test and subject were calculated based on the HC group’s means and standard deviations (SD). Expected z-scores adjusted for age, sex, and education for each test and subject were calculated based on a multiple regression analysis performed in the HC group^1^. Subjects were classified as having MCI as previously described^14^; briefly, if the actual z-score for a test was ≥ 1.5 lower than the expected score in at least two tests in one domain or in one test per domain in at least two domains. This classification scheme fits level I criteria for the diagnosis of MCI in PD according to the Movement Disorder Society Task Force^58^. Composite z-scores for each cognitive function were calculated as the mean of the difference between actual and expected z-scores of all tests within that function.

### MRI acquisition and preprocessing

Three-dimensional structural T1-weighted images, functional resting-state images (training sample: 10-minute duration, 300 volumes; validation sample: 6 minutes, 180 volumes), and FLAIR images were acquired with the same 3 T Siemens MRI scanner, as previously described^14^. Basic functional image preprocessing, using AFNI (http://afni.nimh.nih.gov/afni) tools, included: discarding the first 5 volumes to allow magnetization stabilization, despiking, motion correction, grand-mean scaling, linear detrending, and bandpass filtering (maintaining frequencies between 0.01 and 0.1 Hz).

### Noise correction

In order to remove the effects of head motion and other non-neural sources of signal variation from the functional data, we used an anatomical component-based noise correction method (aCompCor)^63^. In this step, the binary WM and ventricular cerebrospinal fluid (CSF) masks generated with FreeSurfer (version 5.1, http://surfer.nmr.harvard.edu) were linearly registered to native functional space using FSL (release 5.0.9, http://www.fmrib.ox.ac.uk/fsl), and subsequently eroded by applying a threshold of 0.9 (WM mask) and 0.3 (CSF). The time series of all voxels contained in both masks combined were extracted, and the corresponding 10 first principal components were calculated (MATLAB, version 2015b, http://www.mathworks.com/products) and stored to be used as covariates during network computation (see below).

As a quality control measure to assess the efficacy of aCompCor in reducing the relationship between signal variation and motion, we performed correlations between framewise head displacement (calculated as in ref. 64) and percent signal change (derivative of root mean square variance over voxels) before and after regressing the 10 aCompCor components and the six motion parameters out of the functional data^65^. Mean Pearson correlation coefficient was 0.23 (SD = 0.17) before noise correction, and 0.02 (SD = 0.10) afterwards (p < 0.001, repeated-samples t-test), and did not differ significantly between HC and PD subjects (p = 0.490).

### Brain parcellation, time series extraction, and functional network computation

Within the framework of connectomics, the brain is represented as a collection of nodes, linked by edges. Nodes should ideally be defined by regions with coherent patterns of connectivity^66^. The Brainnetome Atlas^67^, a cross-validated connectivity-based parcellation scheme, was used to segment the functional data sets into 210 cortical and 36 subcortical cerebral gray matter regions. After non-linearly transforming the functional data sets to MNI space at 3 × 3 × 3 mm^3^ voxel size with SPM (http://www.fil.ion.ucl.ac.uk/spm/), the first eigenvariates of the time series of all voxels included in each mask were extracted^68^.

Individual brain networks were defined as the 210 cortical + 36 subcortical brain regions as nodes, and the partial correlation between the first eigenvariates of nodes *i* and *j* taken as their interconnecting edges. The 10 principal components from CSF and WM time series as well as the six motion parameters obtained from the motion correction step were used as covariates of no interest in the partial correlation computation. The resulting undirected, fully connected and weighted networks thus consisted of 246 nodes and 30,135 unique edges.

### Classification algorithm

Initially, PD patients in the training sample were divided into PD-MCI (n = 27) and PD-nonMCI (n = 43) groups based on neuropsychological testing performance. LOOCV was used to avoid circularity in subject classification (PD-MCI *vs.* PD-nonMCI). LOOCV excludes one subject for testing whereas the remaining n-1 subjects are used as a training set. This process is repeated once for each subject, enhancing the generalization power of the classifier and preventing over-fitting on the small sample dataset. LOOCV was used to assess classification performance as it returns an almost unbiased estimate of the probability of test error of a classification^69^.

A critical procedure in machine-learning, called *feature selection*, is the identification of variables that are useful for prediction^70^. Another important step, *regularization*, reduces model overfitting by adding a penalty to complex solutions, and increases generalization. L1 and L2 regularization are typically used as they limit the magnitude of model weights by adding a penalty for weights to the model error function. L1 regularization uses the sum of the absolute values of the weights, whereas L2 regularization uses the sum of their squared values. Since L1 regularization leads to sparse solutions by forcing the fitted coefficients of some features to be zero, it performs feature selection as well as regularization^71^. Nonetheless, in the presence of highly correlated features, L1 regularization will choose a few features and set the rest to zero, regardless of their relevance^72^, potentially leading to inconsistent models^73^.

**Figure 4:**
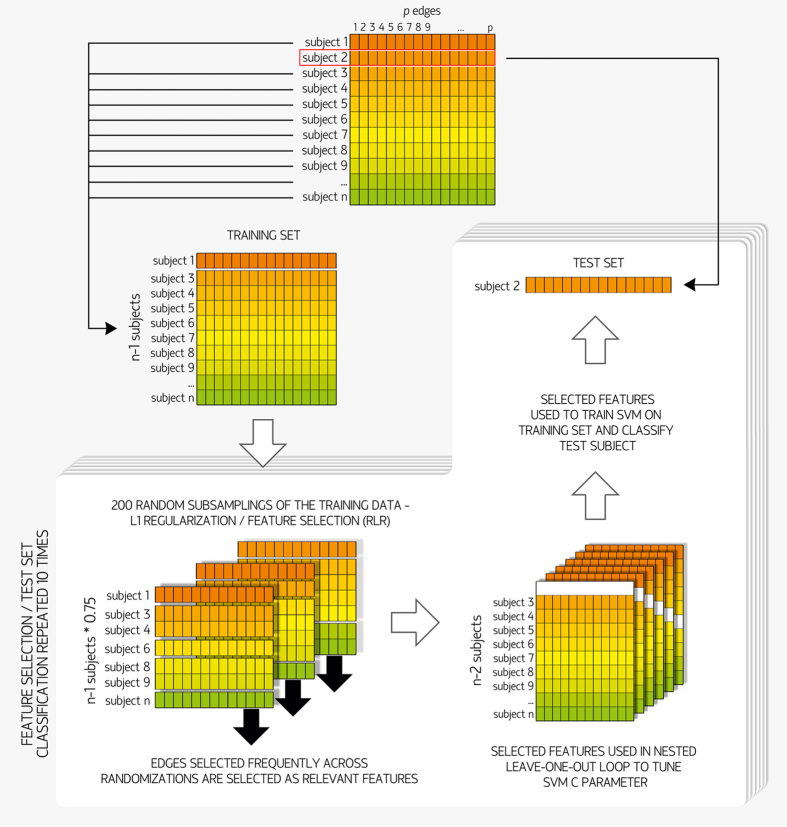
Machine-learning algorithm. Initially, the connectivity matrices of all 70 PD subjects were vectorized, and entered into the 70 leave-one-out cross-validation (LOOCV) iterations. For each iteration, one subject was defined as the test set, whereas the remaining 69 subjects made up the training set. Each training set was then fed into the 10 repeats of the feature selection procedure, *randomized logistic regression* (RLR). In each repeat, the features selected were used in a nested LOOCV loop (69 iterations) to tune the support vector machine (SVM) C parameter, and then to train a linear SVM on the training set. The resulting classifier model was then used to classify the corresponding test subject.

One way to overcome this limitation of L1 regularization in obtaining a stable feature selection is through *stability selection*, which combines data subsampling with selection algorithms^73^,^74^. We performed feature selection through a stability selection technique called *randomized logistic regression* (RLR) with the scikit-learn library (http://scikit-learn.org)^75^, implemented in Python. RLR combines L1-penalized models for classification with randomization of the data. Specifically, for each of 200 iterations, a random subsample of 75% of the data was fitted to an L1-penalized logistic regression model, and features with non-zero coefficients were extracted (see Fig. 4). As relevant features are expected to be selected in most iterations, RLR assigns higher scores to features that are repeatedly chosen across different subsamples, and finally extracts the features with high scores. RLR was applied to each training set of the LOOCV loop, obtaining a set of features for each test subject.

For each of a total of 10 RLR repetitions per training set, the selected features were extracted from the original connectivity matrices, and entered in the classification procedure (PD-MCI *vs.* PD-nonMCI), performed using the LibLINEAR Linear Support Vector Classification^76^ from scikit-learn.

The support vector machine (SVM) *C* parameter (amount of regularization) was defined through a nested LOOCV procedure^77^,^78^,^79^ using grid search (range = 10^−3^, 10^−2^, 10^−1^, …, 10^2^) and L2 regularization. The optimal C parameter was selected as the one with the highest mean accuracy across the iterations of the inner loop. This parameter and the selected features were then used to train the SVM model on the training set (69 samples); this model, in turn, was used to classify the test subject. Therefore, the test set was never part of the process to select the features, or to train the SVM model, to be used in its classification.

Classification in SVM is achieved by constructing the hyper-plane that has the largest distance to the nearest training data points of any class, since larger margins are associated with lower generalization error of the classifier^80^. Linear SVM is an implementation of SVM for the case of a linear kernel. Due to the different class sizes, class weights (inversely proportional to class frequencies) were applied. The whole procedure (feature selection and SVM) was repeated 10 times per training set in order to assess the stability of the results. Reported accuracies and areas under the receiver operating characteristic curves (AUC) therefore refer to the mean values obtained across 10 feature selection/classification repeats performed at each outer LOOCV iteration.

Several of the network edges selected as features displayed a high stability across RLR repeats and across training sets (LOOCV iterations). One-hundred-ninety-three edges were selected in at least one RLR repeat, in at least one training set. Of these, 89 edges were selected in at least one training set in all 10 RLR repeats. Among these 89 edges, 21 were consistently selected in at least 80% of the training sets in all RLR repeats (Fig. 2). As we expect relevant features to be consistent across the LOOCV iterations, we used this latter threshold to define the set of edges that were consistently selected; these edges were then used to train the model used to classify the validation sample, as well as for correlation analyses. In the validation step, the 21 edges described above were used to train a SVM on the entire training sample (70 patients). The trained model was then used to classify the 25 patients from the validation sample.

### Characterization of intergroup connectome differences and their clinical correlates

In order to test for differences in functional connectivity strength between groups, we used *network-based statistics* (NBS)^81^. NBS is a technique that aims to identify *connected components*, consisting of a set of topologically neighboring edges that display statistical effects above a predetermined threshold. Permutation testing is used to define the component-wise p-value based on the sizes (number of edges) of the components that survive an initial component-defining threshold. Pairwise comparisons were done with a threshold of F = 11. The Monte Carlo method with 10,000 permutations (shuffling group membership) was performed to establish significance. Components were considered statistically significant if they displayed a p value ≤ 0.0167 (to account for the three intergroup comparisons performed). As the choice of the initial threshold in NBS is often arbitrary and can affect the sensitivity of the method^81^, we performed comparisons using more liberal F-thresholds (6, 7, 8, 9, 10) if the initial parameter yielded no significant results, to assess the presence of weaker effects.

Other statistical comparisons involving functional connectivity variables (comparison of mean connectivity values, analysis of regional distribution of altered connections, multiple linear regression) were also performed through the Monte Carlo method, with 10,000 permutations. Two-tailed p-values were calculated as the proportion of values in the null distribution more extreme than those observed in the actual model, corrected for multiple testing through control of the false-discovery rate (FDR)^82^ to 5%, when appropriate.

## Additional Information

**How to cite this article:** Abós, A. *et al*. Discriminating cognitive status in Parkinson’s disease through functional connectomics and machine learning. *Sci. Rep.* **7**, 45347; doi: 10.1038/srep45347 (2017).

**Publisher's note:** Springer Nature remains neutral with regard to jurisdictional claims in published maps and institutional affiliations.

## Supplementary Material

Supplementary Results

## Figures and Tables

**Table 1:** Sociodemographic, clinical and head motion by group.

	HC (n = 38)	PD-nonMCI (n = 43)	PD-MCI (n = 27)	stat/p
Age	63.3(10.3)	63.6(9.5)	66.5(11.1)	0.915/0.404
Sex (female)	17	19	10	0.457χ/0.796
Years of education	11.5(4.5)	11.9(5.2)	10.0(5.5)	1.277/0.283
Hand dominance (right)	36	41	27	1.406χ/0.495
UPDRS	—	14.6(7.2)	16.8(11.0)	0.931†/0.358
HY (1:1.5:2:2.5:3)	—	11:5:22:3:2	4:0:15:3:5	7.618χ /0.107
Disease duration	—	5.9(4.0)	10.1(7.2)	**2.797†/0.008**
LEDD	—	646.7(434.2)	950.7(555.2)	**2.558†/0.013**
MMSE	29.7(0.5)	29.4(0.7)	28.6(1.5)	**14.946‡/0.001**
Mean attention/working memory	0.06(0.75)	0.13(0.68)	0.31(0.75)	1.016/0.366
Mean executive function	0.04(0.64)	−0.02(0.65)	−1.69(1.80)	**26.253/<0.001**
Mean memory	−0.01(0.56)	−0.06(1.04)	−0.91(0.86)	**24.150/<0.001**
Mean VS/VP	−0.02(0.8)	−0.23(0.62)	−1.53(1.05)	**15.034/<0.001**
Head rotation (degrees)	0.03(0.01)	0.04(0.02)	0.04(0.02)	**6.775/0.002**
Head translation (mm)	0.08(0.04)	0.08(0.04)	0.08(0.03)	0.090/0.914

*HC:* healthy controls; *PD-nonMCI:* Parkinson’s disease patients without mild cognitive impairment; *PD-MCI*: Parkinson’s disease patients with mild cognitive impairment. *UPDRS:* Unified Parkinson’s disease rating scale, motor section; *HY:* Hoehn and Yahr scale; *Disease duration:* duration of motor symptoms, in years; *LEDD:* levodopa equivalent daily dose, in mg; *MMSE:* mini-mental state examination; *VS/VP:* visuospatial/visuoperceptual score. Head motion parameters refer to mean interframe values. Test stats refer to F, Student’s t (†), Pearson’s chi-square (χ), or Kruskal-Wallis chi-squared (‡). Head motion parameters refer to mean interframe values. Post-hoc tests showed that MMSE, mean executive function, mean memory, and mean VS/VP scores were significantly lower in PD-MCI than both PD-nonMCI and HC (p < 0.05, FDR-corrected), with no significant differences between the latter. Head rotation was significantly lower in HC than both PD-nonMCI and PD-MCI (p < 0.05, FDR-corrected), with no significant differences between the latter.
