# Supplementary material for: Discriminating cognitive status in Parkinson’s disease through functional connectomics and machine learning
Source: Sci Rep. 2017 Mar 28;7:45347. doi: 10.1038/srep45347 (PMC5368610; doi:10.1038/srep45347)
Supplement: Supplementary Results [file srep45347-s1.pdf]

Discriminating cognitive status in Parkinson's disease through functional connectomics and machine learning

Alexandra Abós, Hugo C. Baggio, Bàrbara Segura, Anna I. García-Díaz, Yaroslau Compta, Maria José Martí, Francesc Valldeoriola, Carme Junqué

SUPPLEMENTARY DATA

Supplementary Table 1. Sociodemographic, clinical and head motion comparison between PD-nonMCI and PD-MCI subjects matched for age and motor disease severity.

	PD-nonMCI (n=26)	PD-MCI (n=27)	stat/p
Age	65.9(9.3)	66.5(11.1)	0.212/.833
Sex (female)	9	10	0.034 χ/.854
Years of education	11.1(4.7)	10(5.5)	0.770/.445
Hand dominance (right)	25	27	1.058 χ /.304
UPDRS	16.3(7.2)	16.8(11)	0.200/.842
HY (1:1.5:2:2.5:3)	1:4:17:2:2	4:0:15:3:5	7.394χ /.116
Disease duration	7.5(4.1)	10.1(7.2)	1.648/.107
LEDD	850.5(382.9)	950.7(555.2)	0.762/.450
MMSE	29.3(0.8)	28.6(1.5)	257.0‡/.078
Mean attention/working memory	0.14(0.67)	0.32(0.74)	1.041/.301
Mean executive function	-0.03(0.64)	-1.63(1.81)	4.646/<.001
Mean memory	-0.19(1.11)	-0.91(0.86)	4.497/<.001
Mean VS/VP	-0.31(0.59)	-1.53(1.05)	2.987/.004
Head rotation (degrees)	0.04(0.02)	0.04(0.02)	0.199/.843
Head translation (mm)	0.09(0.05)	0.08(0.03)	1.112/.272

PD-nonMCI: Parkinson's disease patients without mild cognitive impairment; *PD-MCI*: Parkinson's disease patients with mild cognitive impairment. *UPDRS*: Unified Parkinson's disease rating scale, motor section; *HY*: Hoehn and Yahr scale; *Disease duration*: duration of motor symptoms, in years; *LEDD*: levodopa equivalent daily dose, in mg; *MMSE*: mini-mental state examination; *VS/VP*: visuospatial/visuoperceptual score. Head motion parameters refer to mean interframe values. Test stats refer to Student's t, Pearson's chi-square (χ), or Mann-Whitney's U (‡).

Supplementary Figure 1. Mean connectivity matrices according to group.

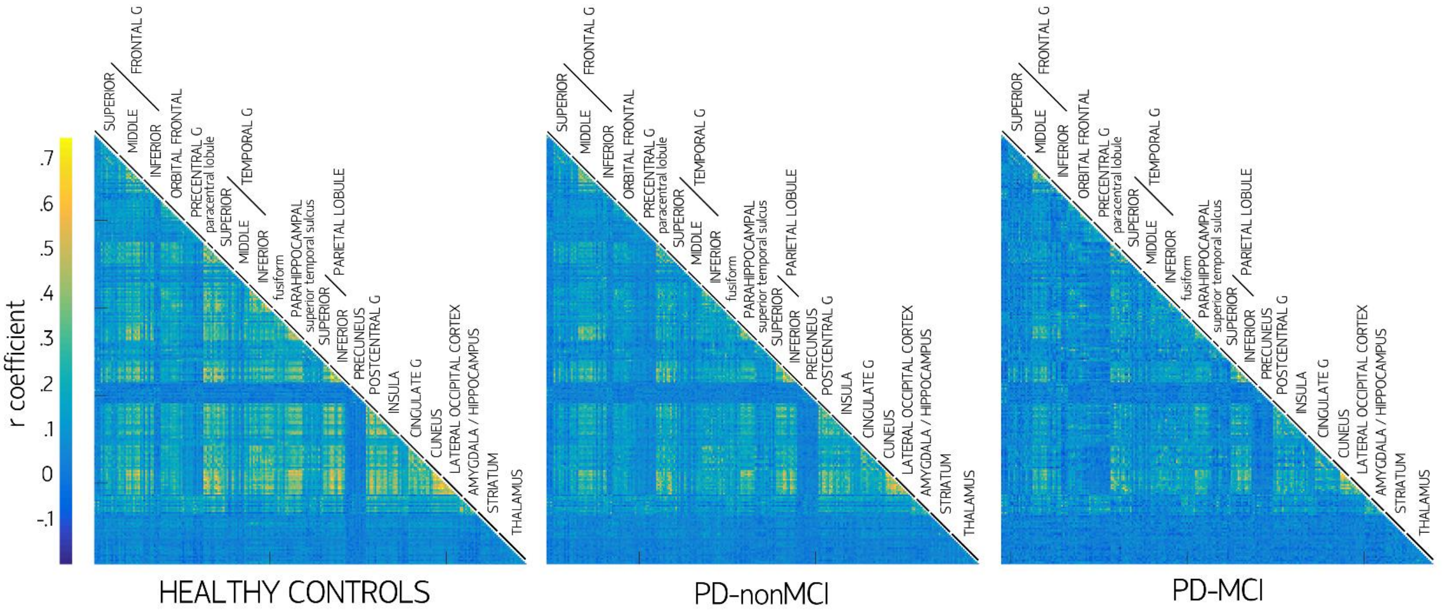

Mean strength of connectivity between the 246 Brainnetome atlas nodes, according to group. *PD-nonMCI*: Parkinson's disease patients without mild cognitive impairment. *PD-MCI:* Parkinson's disease patients with mild cognitive impairment.

Supplementary Figure 2. Global mean connectivity strength by group.

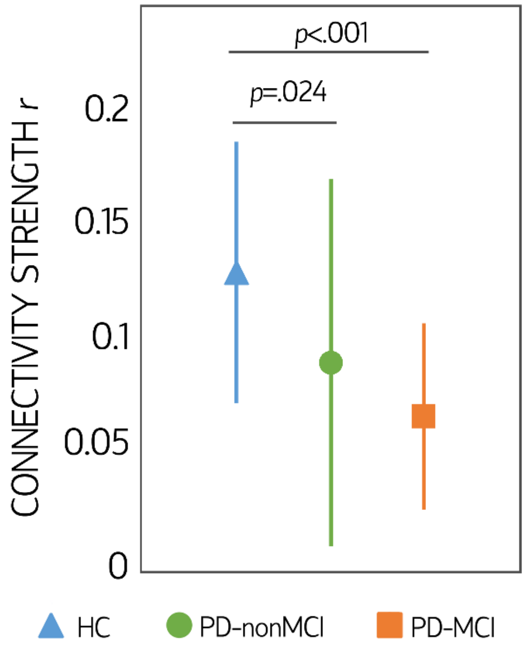

Mean whole-connectome connectivity strength according to group. Error bars indicate one standard deviation. *HC:* healthy controls; *PD-nonMCI:* Parkinson's disease patients without mild cognitive impairment. *PD-MCI:* Parkinson's disease patients with mild cognitive impairment.

Supplementary Figure 3. Distribution of edges identified in intergroup comparisons and feature selection.

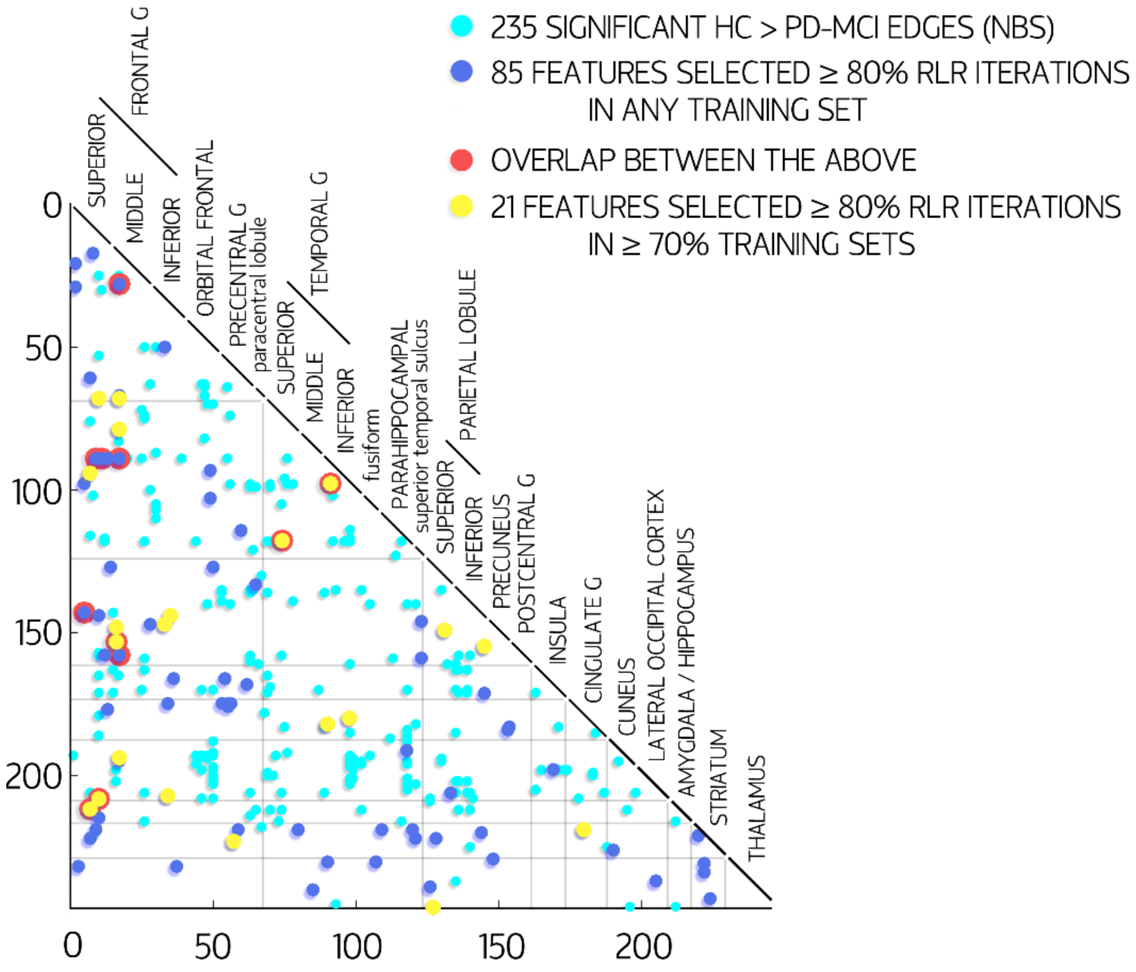

Light cyan circles represent the 235 edges comprised in the component of significantly reduced functional connectivity strength in Parkinson's disease patients with mild cognitive impairment (PD-MCI) compared with controls (HC) using network-based statistics (NBS). *Dark blue* circles represent the 85 edges selected as features in at least 80% of the iterations of the feature selection procedure (RLR – randomized logistic regression), in at least one training set in the leave-one-out cross-validation loop. *Red circles* indicate where light and blue circles overlap. *Yellow circles* indicate the 21 edges most consistently selected as features through RLR.

Supplementary Figure 4. Distribution of nodes overrepresented in the component of reduced connectivity strength in Parkinson's disease patients with mild cognitive impairment compared with healthy controls.

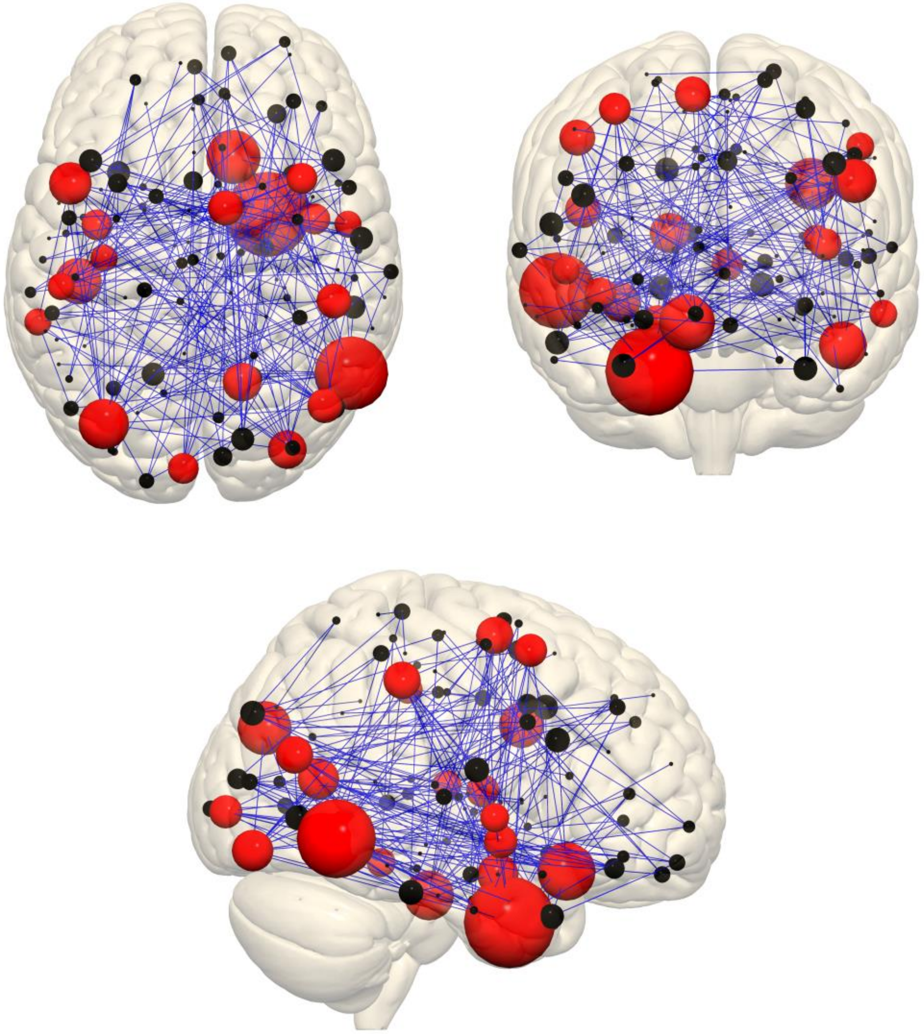

NODE LABEL	NUMBER OF ALTERED CONNECTIONS
PhG_R_6_5	22
ITG_R_7_5	19
OrG_R_6_5	14
IPL_L_6_1	13
ITG_L_7_1	12
PrG_L_6_6	11
Cun_R_5_5	10
OcG_R_4_4	10
Amyg_R_2_1	10
SFG_R_7_5	9
IPL_R_6_1	9
IPL_R_6_3	9
MFG_R_7_6	8
INS_R_6_4	8
INS_L_6_5	8
Cun_L_5_3	8
STG_R_6_3	7
ITG_L_7_6	7
IPL_L_6_3	7
INS_L_6_1	7
MFG_L_7_2	6
IFG_R_6_1	6
STG_L_6_1	6
STG_R_6_1	6
ITG_R_7_7	6
PoG_R_4_2	6
Cun_L_5_4	6
Cun_R_5_4	6
OcG_R_4_2	6
sOcG_R_2_1	6
MFG_L_7_6	5
OrG_R_6_3	5
CG_L_7_5	5
Cun_R_5_3	5

Left: schematic representation of the component consisting of 235 edges considered significantly different between groups (p<.05, family-wise error corrected). Brain nodes are scaled according to the number of edges in the significant component to which they are connected. Nodes with more altered connections than predicted by chance (compared with randomly reshuffling the 235 altered connections across the brain network 10,000 times; p<.05, with false-discovery rate control) are colored in red. *Right:* Labels of the nodes overrepresented in the altered component are shown, along with the number of altered edges connected to them. Brain plots were created with Surf Ice (https://www.nitrc.org/projects/surfice/). See also Supplementary Table 2

Supplementary Table 2. Sociodemographic, clinical and head motion comparison between PD-nonMCI and PD-MCI in the validation sample.

	PD-nonMCI (n=8)	PD-MCI (n=17)	stat/p
Age	60.4(10.4)	69.9(7.6)	2.305/.031
Sex (female)	2	3	2.252 χ/.133
Years of education	11.2(5.3)	11.8(5.7)	0.247/.807
Hand dominance (right)	8	17	-
UPDRS	14.5(8.0)	17.5(7.0)	0.883/.387
HY (1:2:3)	0:6:2	2:14:0†	5.100χ /.078
Disease duration	5.6(4.7)	8.0(3.4)	1.304/.206
LEDD	553.2(429.4)	748.6 (195.5)	1.145/.265
MMSE	29.5(0.5)	27.5(2.5)	33.0‡/.027
Mean attention/working memory	0.20(0.79)	0.06(0.71)	0.419/.679
Mean executive function	-0.33(0.68)	-2.16(1.78)	2.808/.023
Mean memory	-0.02(0.90)	-1.74(1.12)	4.109/<.001
Mean VS/VP	-0.10(0.55)	-1.40(0.68)	5.078/<.001
Head rotation (degrees)	0.05(0.02)	0.06(0.04)	1.162/.257
Head translation (mm)	0.11(0.05)	0.12(0.08)	0.347 /.732

PD-nonMCI: Parkinson's disease patients without mild cognitive impairment; *PD-MCI*: Parkinson's disease patients with mild cognitive impairment. *UPDRS*: Unified Parkinson's disease rating scale, motor section; *HY*: Hoehn and Yahr scale († one missing value); *Disease duration*: duration of motor symptoms, in years; *LEDD*: levodopa equivalent daily dose, in mg; *MMSE*: mini-mental state examination; *VS/VP*: visuospatial/visuoperceptual score. Head motion parameters refer to mean interframe values. Test stats refer to Student's t, Pearson's chi-square (χ), or Mann-Whitney's U (‡).

Supplementary Table 3. Brainnetome atlas nodes and corresponding anatomical regions.

**Cingulate**
CG_L(R)_7_1, CG_L(R)_7_2, CG_L(R)_7_3, CG_L(R)_7_4, CG_L(R)_7_5, CG_L(R)_7_6, CG_L(R)_7_7
**Frontal**
SFG_L(R)_7_1, SFG_L(R)_7_2, SFG_L(R)_7_3, SFG_L(R)_7_4, SFG_L(R)_7_5, SFG_L(R)_7_6, SFG_L(R)_7_7, MFG_L(R)_7_1, MFG_L(R)_7_2, MFG_L(R)_7_3, MFG_L(R)_7_4, MFG_L(R)_7_5, MFG_L(R)_7_6, MFG_L(R)_7_7, IFG_L(R)_6_1, IFG_L(R)_6_2, IFG_L(R)_6_3, IFG_L(R)_6_4, IFG_L(R)_6_5, IFG_L(R)_6_6, OrG_L(R)_6_1, OrG_L(R)_6_2, OrG_L(R)_6_3, OrG_L(R)_6_4, OrG_L(R)_6_5, OrG_L(R)_6_6, PrG_L(R)_6_1, PrG_L(R)_6_2, PrG_L(R)_6_3, PrG_L(R)_6_4, PrG_L(R)_6_5, PrG_L(R)_6_6, PCL_L(R)_2_1
**Insula**
INS_L(R)_6_1, INS_L(R)_6_2, INS_L(R)_6_3, INS_L(R)_6_4, INS_L(R)_6_5, INS_L(R)_6_6
**Medial temporal**
FuG_L(R)_3_1, FuG_L(R)_3_2, FuG_L(R)_3_3, PhG_L(R)_6_1, PhG_L(R)_6_2, PhG_L(R)_6_3, PhG_L(R)_6_4, PhG_L(R)_6_5, PhG_L(R)_6_6, Amyg_L(R)_2_1, Amyg_L(R)_2_2, Hipp_L(R)_2_1, Hipp_L(R)_2_2
**Occipital**
MVOcC_L(R)_5_1, MVOcC_L(R)_5_2, MVOcC_L(R)_5_3, MVOcC_L(R)_5_4, MVOcC_L(R)_5_5, LOcC_L(R)_4_1, LOcC_L(R)_4_2, LOcC_L(R)_4_3, LOcC_L(R)_4_4, LOcC_L(R)_2_1, LOcC_L(R)_2_2
**Parietal**

SPL_L(R)_5_1, SPL_L(R)_5_2, SPL_L(R)_5_3, SPL_L(R)_5_4, SPL_L(R)_5_5, IPL_L(R)_6_1, IPL_L(R)_6_2, IPL_L(R)_6_3, IPL_L(R)_6_4, IPL_L(R)_6_5, IPL_L(R)_6_6, Pcun_L(R)_4_1, Pcun_L(R)_4_2, Pcun_L(R)_4_3, Pcun_L(R)_4_4, PoG_L(R)_4_1, PoG_L(R)_4_2, PoG_L(R)_4_3, PoG_L(R)_4_4
**Basal ganglia**
BG_L(R)_6_1, BG_L(R)_6_2, BG_L(R)_6_3, BG_L(R)_6_4, BG_L(R)_6_5, BG_L(R)_6_6
**Temporal**
STG_L(R)_6_1, STG_L(R)_6_2, STG_L(R)_6_3, STG_L(R)_6_4, STG_L(R)_6_5, STG_L(R)_6_6, MTG_L(R)_4_1, MTG_L(R)_4_2, MTG_L(R)_4_3, MTG_L(R)_4_4, ITG_L(R)_7_1, ITG_L(R)_7_2, ITG_L(R)_7_3, ITG_L(R)_7_4, ITG_L(R)_7_5, ITG_L(R)_7_6, ITG_L(R)_7_7, pSTS_L(R)_2_1, pSTS_L(R)_2_2
**Thalamus**
Tha_L(R)_8_1, Tha_L(R)_8_2, Tha_L(R)_8_3, Tha_L(R)_8_4, Tha_L(R)_8_5, Tha_L(R)_8_6, Tha_L(R)_8_7, Tha_L(R)_8_8

CG: cingulate gyrus; SFG: superior frontal gyrus; MFG: middle frontal gyrus; IFG: inferior frontal gyrus; OrG: orbital gyrus; PrG: precentral gyrus; PCL: paracentral lobule; INS: insula; FuG: fusiform gyrus; PhG: parahippocampal gyrus; Amyg; amygdala; Hipp: hippocampus; MVOcC: medioventral occipital cortex; LOcC: lateral occipital cortex; SPL: superior parietal lobule; IPL: inferior parietal lobule; Pcun: precuneus; PoG: postcentral gyrus; BG: basal ganglia; STG: superior temporal gyrus; MTG: middle temporal gyrus; ITG: inferior temporal gyrus; pSTS: posterior superior temporal sulcus; Tha: thalamus. (see also http://cercor.oxfordjournals.org/content/26/8/3508.full.pdf)
